# COVID-19 vaccinations among German university students: The role of fear of COVID-19 and risk assessment

**DOI:** 10.1371/journal.pone.0333082

**Published:** 2025-09-25

**Authors:** Jonas Tögel, Christof Kuhbandner

**Affiliations:** Department of Human Sciences, University of Regensburg, Regensburg, Germany; Regional Health Care and Social Agency of Lodi, ITALY

## Abstract

Studies on the reasons why people have opted for COVID-19 vaccination have shown that a higher fear of COVID-19 and a higher risk assessment of developing a serious illness in case of a SARS-CoV-2 infection correlate positively with higher vaccine uptake. The present study examined whether this also holds true for university students who face a very low risk of developing a serious illness. Fear of COVID-19, risk assessment, and the number of received COVID-19 vaccinations was measured using an online questionnaire which could be completed from December 15, 2022 to the end of January 2023. In total, data from 419 German university students was analyzed. The results show that the vaccination rate among German university students (93.6%) was higher than the vaccination rate of the general population of the same age group. Even in the group of university students who reported having no fear of COVID-19 at all, the vaccination rate was still 83%, and already the experience of low fear and the estimation of low risk led to the vaccination rate rising to almost 100%. Fear of COVID-19 also increased with the willingness to receive additional vaccinations after the initial two doses, and even in this case, already the experience of low fear was associated with a substantial increase in the rate of double-vaccinated individuals who continued to receive further vaccinations. These findings underline the role of fear in people’s decision for COVID-19 vaccination and specifically show that fear can play a substantial role even in groups of people who actually have a very low risk of becoming seriously ill.

## Introduction

During the Covid-19 pandemic, the question whether and why or why not people chose to get vaccinated was the starting point for a vast body of research [1–3]. A multitude of psychological factors that could increase or decrease the likelihood of people to (not) get vaccinated was examined, such as the role of public trust, experienced autonomy, fear of COVID-19, and extrinsic motivation [4–8].

The present study focused on fear of COVID-19 and risk-assessment, therefore the vast body of research that examines the relationship between fear of COVID-19 and risk assessment and people’s willingness to get vaccinated against Covid-19 is particularly relevant. Some studies found that fear of COVID-19 did not predict vaccine uptake [9] or that fear of Covid-19 positively correlated with vaccine hesitancy [10]. However, the vast majority of the studies points towards the tendency that a higher degree of fear correlates positively with a higher vaccine uptake [11–17]. The same is true for risk assessment where studies have shown that a higher risk assessment correlates positively with a higher vaccine uptake [18–21], with the effect of fear of Covid-19 being stronger than the effect of risk assessment.

Taken together, a clear pattern of findings seems to be emerging: The higher the fear of COVID-19 and the higher the risk assessment, the more likely people are to get vaccinated against COVID-19. However, previous studies have examined the relationship between fear of COVID-19, risk assessment, and vaccine uptake almost exclusively at the level of the overall population, raising the question of whether this pattern actually applies equally to all subgroups. In view of the fact that younger people [22,23] and people with higher education and intelligence [24] only rarely become seriously ill with a SARS-CoV-2 infection, the group of university students in particular could represent an exceptional group in which the willingness to be vaccinated is not influenced by fear of COVID-19 and risk assessment.

The main aim of the present study was therefore to investigate whether fear of COVID-19 and risk assessment also play a role in the group of university students as to whether people get vaccinated. To investigate this question, the fear of COVID-19 and the estimated risk of having to be treated in hospital or in an intensive care unit in the event of infection were measured in a large sample of university students, as well as the number of vaccinations someone had received. By determining not only whether someone has been vaccinated at all, but also the exact number of Covid-19 vaccinations, it was additionally possible to investigate another question that has not yet been addressed in previous studies, namely whether fear and risk assessment are not only associated with the decision whether someone gets vaccinated at all, but also how often someone gets vaccinated.

## Method

### Participants

Data collection started on December 15, 2022, and ended on January 31, 2023. By that time, the vaccination campaign in Germany had already concluded, and peak vaccination rates in the relevant age group had been reached in the spring of 2022 [25]. Participant recruitment was conducted primarily through large-scale university lectures (with study advertisement via QR-coded PowerPoint slides) and social media platforms frequented by the target demographic (featuring brief study summaries with QR codes). Due to the nature of this approach, precise response rates could not be calculated as the total exposure to recruitment invitations remained unmeasurable. If required, university students received course credit for participation.

The sample size was determined by including all individuals participating in the study by the end of the data collection period in the data analysis. Only individuals currently enrolled in a university were eligible for inclusion in the study. A total of 428 university students took part in the survey. Nine participants did not provide information on their vaccination status. The final sample consisted of 419 participants (333 women, 85 men, 1 without specification). The mean age was 21.5 years (ranging from 18 to 44 years, *SD* = 3.1). All participants stated that they lived in Germany, with 91.2% living in Bavaria. The study was conducted in accordance with the Helsinki Declaration and the University Research Ethics Standards of the University of Regensburg. All participants provided informed consent prior to filling out the questionnaire. In Germany, these types of psychological studies do not require ethical approval of an Ethics Committee (see https://www.dfg.de/foerderung/faq/geistes_sozialwissenschaften/).

### Procedure and measures

The study was conducted online via SoSci Survey [26]. The data reported in this study were part of a more comprehensive questionnaire on the motivational background of mask wearing. Fear of Covid-19 was measured using the statement “I am afraid of Covid-19” which was rated on a Likert scale ranging from 1 (“does not apply to me at all”), 2 (“mostly does not apply to me”), 3 (“mostly applies to me”) to 4 (“fully applies to me”). Similar single-item measurements to assess fear of COVID-19 have been used in previous research [12]. The estimated risk associated with a SARS-CoV-2-infection was measured with two items with an open response format: “Please estimate: How many out of 10,000 people of your age who become infected with SARS-CoV-2 need to be treated in hospital”, followed by the request “Please enter the number here:... out of 10,000 infected people of my age need to be treated in the hospital”, and “Please estimate: How many out of 10,000 people of your age who become infected with SARS-CoV-2 need to be treated in intensive care”, followed by the request “Please enter the number here:... out of 10,000 infected people of my age need to be treated in intensive care”. Similar measurements to assess estimated risk have been used in previous research [27]. Values that were more than five standard deviations above the respective means were considered to be presumably incorrect entries and were therefore excluded from the analyses (5 values). Vaccination status was measured with the question “Please indicate whether you have been vaccinated against COVID-19, and if so, how many vaccinations you have received”. The possible answers were: “no answer”, “not vaccinated”, “one vaccination”, “two vaccinations”, “three vaccinations”, “four vaccinations”.

### Statistical analysis

Descriptive statistics were used to report vaccination rates, fear of COVID-19, and risk estimation (perceived risks of hospitalization and intensive care treatment due to a SARS-CoV-2 infection). To examine the association between vaccination status (none, 1–2 doses, 3–4 doses) and perceived threat, one-way analyses of variance (ANOVAs) were conducted with vaccination status as the between-subjects factor and fear or risk estimates as dependent variables. Linear and quadratic polynomial contrast analyses were used to assess potential trends across vaccination groups. Additional one-way ANOVAs were performed to assess differences in vaccination and booster uptake by levels of self-reported fear of COVID-19 (none, low, medium, high). Independent samples *t* tests were used to compare vaccination rates between participants with very low risk estimations (hospitalization < 0.1%; intensive care < 0.05%) and all others. Finally, binary logistic regression analyses were conducted to examine the predictive value of fear of COVID-19 and perceived risk on vaccination and booster uptake. Fear (none vs any fear) and estimated risk (very low vs not very low) were included as categorical predictors. Odds ratios (ORs) with 95% confidence intervals (CIs) are reported. Statistical significance was set at *p* < .05 (two-tailed) for all tests. All analyses were conducted using SPSS, version 25 (IBM Corp).

## Results

**Fig 1:**
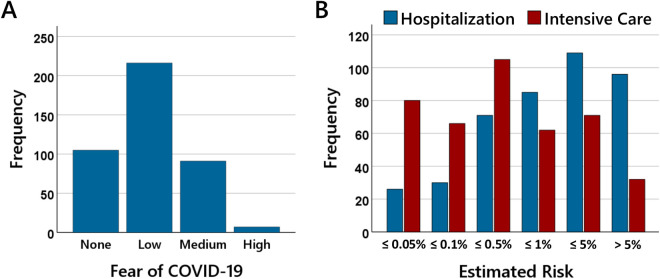
Fear of COVID-19 and risk estimation. The bars in (A) show the number of participants that stated to have none, low, medium, or high fear of COVID-19. The bars in (B) show the numbers of participants who estimated the risk of hospitalization (blue bars) and the risk of intensive care treatment (red bars) associated with a SARS-CoV-2-infection to be ≤ 0.05%, ≤ 0.1%, ≤ 0.5%, ≤ 1%, ≤ 5%, or > 5%.

Across all participants, the vaccination rate was 93.6%. Of the people who stated that they had been vaccinated, 1.0% stated that they had been vaccinated once, 22.7% that they had been vaccinated twice, 73.5% that they had been vaccinated three times, and 2.8% that they had been vaccinated four times. Fig 1 shows the participants’ ratings for the fear of COVID-19 and the estimated risk of having to be treated in hospital and having to be treated in intensive care in the event of a SARS-CoV-2-infection.

25.1% of the participants stated they had no fear of COVID-19, 51.6% stated they had low fear of COVID-19, 27.1% stated that they had medium fear of COVID-19, and only 1.7% stated they had high fear of COVID-19. 13.4% of the participants estimated that out of 10,000 infected people of their age, 10 or fewer people (risk ≤ 0.1%) would need to be hospitalized, 16.9% that between 10 and 50 people (risk > 0.1% and ≤ 0. 5%) would need to be hospitalized, and 23% that between 50 and 100 people (risk > 0.5% and ≤ 1.0%) would need to be hospitalized. This means that 50 percent of the participants stated that the risk of hospitalization associated with a SARS-CoV2-infection was one percent or less. 23.0% stated that the risk of hospitalization was more than five percent. 19.1% of the participants estimated that out of 10,000 infected people of their age, 5 or fewer people (risk ≤ 0.05%) would need intensive care treatment, 15.8% that between 5 and 10 people (risk > 0.05% and ≤ 0.1%) would need intensive care treatment, and 25.1% that between 10 and 50 people (risk > 0.1% and ≤ 0.5%) would need intensive care treatment. This means that 60 percent of participants stated that the risk of needing intensive care treatment associated with a SARS-CoV2-infection was 0.5 percent or less. Only 24.8% stated that the risk of needing intensive care treatment was more than one percent.

**Fig 2:**
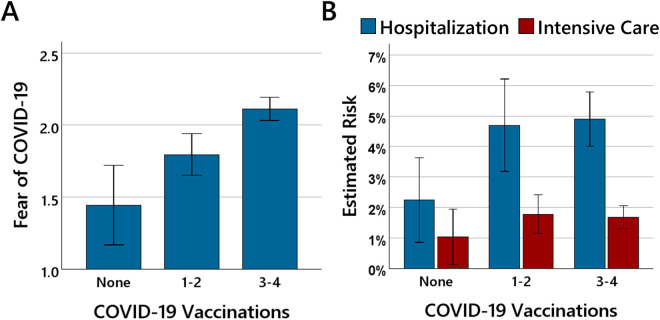
Fear of COVID-19 and risk estimation as a function of vaccination status. The bars in (A) show the mean fear of COVID-19 and the bars in (B) the mean estimated risks of hospitalization and treatment in intensive care associated with a SARS-CoV-2-infection for participants with none, 1-2 vaccinations, and 3-4 vaccinations. The error bars show 95% confidence intervals.

Next, it was investigated whether participants with different vaccination status differed in the extent of fear of COVID-19 and in the estimated risk associated with a SARS-CoV-2 infection. Fig 2 shows for participants without vaccination, with 1−2 vaccinations, and with 3−4 vaccination the average estimated fear of COVID-19 and the average estimated risks of hospitalization and treatment in intensive care associated with a SARS-CoV-2-infection.

For fear of COVID-19, a one-way ANOVAs with the factor of vaccination status (none vs. 1–2 vs. 3–4) revealed a significant main effect of vaccination status, *F*(2, 416) = 16.09, *p* < .001, *η²* = .07. Polynomial contrast analysis indicated a significant linear trend, *p* < .001, suggesting that fear of COVID-19 increased with the number of vaccinations. The quadratic contrast was not significant, *p* = .871. For risk estimation, the analyses revealed no significant effect, neither for the estimated risk of hospitalization, *F*(2, 414) = 1.50, *p* = .224, *η²* = .01, nor for the estimated risk of treatment in intensive care, *F*(2, 413) = 0.574, *p* = .564, *η²* < .01.

**Fig 3:**
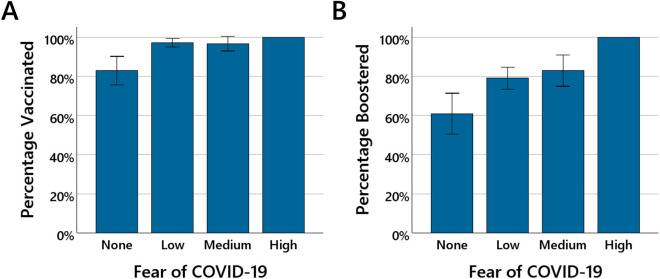
Fear of COVID-19 and vaccination rate. The bars in (A) show the percentage of participants who had decided to be vaccinated and the bars in (B) the percentage of vaccinated participants who had decided to receive more than two vaccinations (i.e., booster vaccinations) as a function of fear of COVID-19 (none, low, medium, high). The error bars show 95% confidence intervals.

Next, it was investigated whether fear of COVID-19 and estimated risk associated with a SARS-CoV-2 infection can be used to predict whether a person has chosen to be vaccinated and, if so, how many vaccinations a person has had. Fig 3 shows for participants who stated that they had no, low, medium, or high fear of COVID-19 the percentage of participants who had decided to be vaccinated and the percentage of vaccinated individuals who chose to receive more than two vaccinations (i.e., booster vaccinations).

The vaccination rate was lower in the group of participants with no fear of COVID-19 (82.9%; *SD* = 37.9), and already the experience of low fear of COVID-19 led to the vaccination rate rising to almost 100 percent (low fear: 97.2%; *SD* = 16.5, medium fear: 96.7%; *SD* = 18.0, high fear: 100%; *SD* = 0.0). A similar picture is observed for the rate of vaccinated people who opted for more than two vaccinations (booster vaccination rate). The booster vaccination rate rose from 60.9% (*SD* = 49.1) in the group of participants with no fear of COVID-19 to 79.0% (*SD* = 40.8) in the group of participants with low fear, and then increased further to 83.0% (*SD* = 37.8) in the group of participants with medium fear and to 100% (*SD* = 0.0) in the group of participants with high fear. For both the vaccination rate and the booster vaccination rate, one-way ANOVAs with the factor of fear of COVID-19 (none vs. low vs. medium vs. high) revealed significant main effects, *F*(3, 415) = 9.43, *p* < .001, *η²* = .06, and *F*(3, 388) = 5.71, *p* < .001, *η²* = .04.

**Fig 4:**
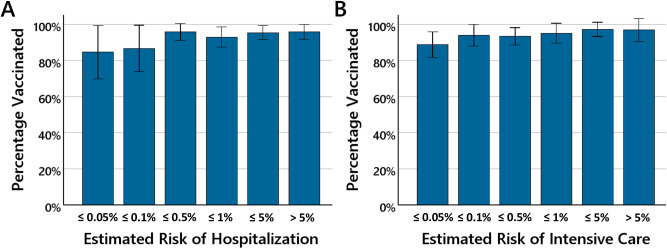
Estimated risk of a SARS-CoV-2-infection and vaccination rate. The bars show the percentage of participants who had decided to be vaccinated as a function of the estimated hospitalization risk (Fig 4A) and the estimated intensive care risk (Fig 4B). The error bars show 95% confidence intervals.

Fig 4 shows the vaccination rates for participants as a function of their risk estimations for hospitalization (Fig 4A) and intensive care treatment (Fig 4B) in the event of a SARS-CoV-2-infection.

In the groups of participants with very low risk estimations (hospitalization: < 0.1%; intensive care: < 0.05%) a tendency towards a lower vaccination rate is observed. In order to increase the power to detect a possible effect, the group of participants was divided into participants with very low risk estimation (hospitalization: < 0.1% and intensive care: < 0.05%; *N* = 54) and all remaining participants. The vaccination rate was lower in the group with very low risk estimation (85.2%; *SD* = 35.9 vs. 95.1%; *SD* = 21.7), the difference was marginally significant, *t*(58.90) = −1.97, *p* = .054. Regarding the booster vaccination ra*t*e, the rates were almost identical between the two groups, (76.1%; *SD* = 43.1 vs. 76.3%; *SD* = 42.6).

Finally, the effects of fear of COVID-19 and of estimated risk of SARS-CoV-2 infection were compared using binary logistic regression analyses with the categorical predictors of fear of COVID-19 (none vs. fear) and estimated risk (very low [hospitalization: < 0.1% and intensive care: < 0.05%] vs. not very low). Prior to analysis, we verified that the sample size was sufficient for the number of predictors included, ensuring an adequate events-per-variable ratio. Multicollinearity was assessed using variance inflation factors (VIF), with all values below 2 indicating no concerning multicollinearity. Influential data points were examined using Cook’s distance, with no cases identified as exerting undue influence on the model.

For both the vaccination rate and the booster vaccination rate, the model was statistically significant, *χ²*(2) = 29.59, *p* < .001 (vaccination rate), and *χ²*(2) = 13.50, *p* = .001 (booster vaccination rate), indicating that the predictors reliably distinguished between vaccination status. For the vaccination rate, the model explained 18.3% (Nagelkerke *R²*) of the variance and correctly classified 93.8% of cases. Both the predictor fear of COVID-19, *B* = −2.06, *SE* = 0.45, Wald = 21.26, *p* < .001, *Exp(B)* = 0.13, 95% CI [0.05, 0.31], and the predictor estimated risk, *B* = −1.19, *SE* = 0.48, *Wald* = 6.14, p = .013, *Exp(B)* = 0.30, 95% CI [0.12, 0.78], were significant predictors. For the booster vaccination rate, the model explained 5.1% (Nagelkerke *R²*) of the variance and correctly classified 76.3% of cases. Only the predictor fear of COVID-19 was significant, *B* = −0.98, *SE* = 0.26, *Wald* = 13.97, *p* < .001, *Exp(B)* = 0.37, 95% CI [0.22, 0.63]. The predictor estimated risk was not significant, *B* = 0.01, *SE* = 0.36, *Wald* < 0.01, *p* = .979.

An analysis of gender effects showed no significant differences for vaccination status or fear of COVID-19. Risk estimation was higher among women *(M = 0.09, SD = 1.07)* than among men *(M =* −*0.30, SD = 0.45).* Because the assumption of equal variances was violated (Levene’s test, *p* < 0.001), a Welch’s *t*-*t*est was conducted, indicating a significant difference, *t*(324.19) = −5.11, *p* < 0.001, *d* = 0.40.

## Discussion

Previous studies have shown that the greater the fear of COVID-19 and the higher the estimated risk associated with a SARS-CoV-2-infection, the more likely people are to choose to get vaccinated against COVID-19 [11–17]. The results of the present study show that this also applies to the group of university students, with the particularly remarkable finding that already the experience of low fear of COVID-19 and the estimation of low risk were associated with the vaccination rate approaching 100 percent. What could also be confirmed for the group of university students is that fear of COVID-19 played a much more important role than the estimated risk of a SARS-CoV-2 infection. The present findings further show that fear of COVID-19 also correlated with a higher willingness to receive additional vaccinations after the initial two doses. Even in this case, already the experience of low fear was sufficient to increase the rate of double-vaccinated individuals who continued to receive further vaccinations from 60 percent (no fear) to 80 percent.

Notably, such an association between fear of COVID-19 and vaccine uptake was observed even though participants had already received their vaccinations. One might have expected that completing vaccination would reduce fear, as individuals could feel protected against COVID-19; however, our data do not support this assumption. One possible explanation is limited confidence in vaccine efficacy. Another possibility is that acquired fear of COVID-19 may be difficult to override through rational beliefs alone. This interpretation is supported by evidence from fear-conditioning research showing that acquired fear responses form robust, amygdala-dependent memory traces that resist modification through rational reappraisal, due to dissociable neural pathways for declarative knowledge and automatic fear responses [e.g., 28]. In the latter case, the observed pattern may reflect a sensitization effect, whereby individuals with higher fear levels are more likely to engage in further protective behaviors, resulting in increased vaccination uptake.

The fact that such a relationship between fear of COVID-19 and the willingness to be vaccinated is even observed among university students is remarkable given that university students are a group of people who only very rarely become seriously ill in the event of a SARS-CoV-2 infection [22,23]. Interestingly, many university students are indeed fully aware of this low risk, as evidenced by the facts that 50 percent of the university students estimated the risk of having to be hospitalized in case of infection to be less than one percent, that 60 percent of the university students estimated the risk of having to be treated in intensive care to be less than 0.5 percent, and that 25 percent of the university students stated that they had no fear of COVID-19 at all.

It is also worth noting that the vaccination rate among university students was very high at 93.6 percent, which is significantly higher than the vaccination rate in the 18–59 age group, which was 81.7 percent at the time of data collection in the region where most of the participants lived. It is particularly surprising that even in the group of students who reported having no fear of COVID-19 at all, the vaccination rate was still 83 percent. This fact is a clear indication that there are additional reasons beyond the fear of COVID-19 and the desire to protect oneself from severe cases of COVID-19 as to why many university students decided to get vaccinated.

There are a number of reasons beyond the fear of COVID-19 and the desire to protect oneself why people have decided to get vaccinated, such as the desire to protect others [29], the existence of social norms and the avoidance of social exclusion [30], and the effectiveness of campaigns to promote vaccination readiness [31]. While all these factors may have contributed to the high vaccination rate in the university student group despite low fear of COVID-19 and low risk assessment, these factors alone cannot explain why the vaccination rate in this group of people was particularly high, because people not studying at universities were equally affected by these factors.

One possible factor that only affected university students is that there were numerous restrictions and requirements that applied specifically to university students in Germany. For example, access to university buildings, to face-to-face events, to university libraries and to canteens was only permitted for people who had been vaccinated, recovered from COVID-19, or had been negatively tested. Unvaccinated university students therefore had to present an officially confirmed negative test result every day [32]. Initially, tests were offered free of charge, but from mid-October 2021, free testing was discontinued for most people so that unvaccinated university students often had to pay for the tests themselves, which led to a considerable financial burden. Some universities went even further and permitted lecturers to only allow vaccinated or recovered students to take part in certain courses so that unvaccinated university students were partially even excluded from face-to-face teaching. Thus, if a university student wanted to remain unvaccinated, severe study-related restrictions and financial losses had to be accepted, which could explain why the vaccination rate among university students was significantly higher than in the general population.

A potential limitation of the study is that 91.2% of participants resided in Bavaria. COVID-19 measures for university students were, however, largely similar across German federal states, suggesting that the findings are likely to be broadly generalizable. At the same time, Bavaria implemented stricter regulations in certain instances, which should be taken into account when interpreting the results. Future studies could extend this work by including more balanced samples across federal states to examine potential effects of regional policy differences.

The present study assessed fear of COVID-19 using a single-item measure. While single-item measures cannot provide internal consistency reliability, they can be appropriate for clearly defined, unidimensional constructs [33,34], and prior research has shown that fear of COVID-19 consistently emerges as a unidimensional factor in psychometric analyses [35]. Our item was designed to capture participants’ overall emotional response to COVID-19 in a concise and pragmatic manner, a format that has also been used in other studies on fear of COVID-19 [12]. Nevertheless, future research could benefit from measuring fear of COVID-19 in a more multifaceted way, enabling the examination of whether specific subcomponents of fear are more strongly associated with vaccine uptake.

This study did not control for other potential factors influencing vaccination behavior, such as personal or family COVID-19 experience, health conditions, political trust, or social norms. These variables were beyond the scope of the present research, which focused specifically on the relationship between fear of COVID-19 and vaccination. Future research could extend this work by incorporating such variables to examine how they interact with fear in predicting vaccination behavior.

Taken together, the findings of the present study show that even in the group of university students who had no fear of COVID-19 at all, the vaccination rate was already over 80 percent, and the rate of vaccinated individuals who received additional doses after the initial two doses was already over 60 percent. Already the experience of low fear of COVID-19 was associated with the vaccination rate approaching nearly 100 percent, and to the rate of vaccinated individuals who received further doses after the initial two doses rising to 80 percent. These findings impressively underline the role of fear in people’s decision for or against vaccination, and specifically show that fear can play a substantial role even in groups of people who have no real reason to be significantly afraid because they have a very low risk of becoming seriously ill. From an ethical perspective, this is an indication of how sensitively topics must be approached that have the potential to trigger fear, as even the evocation of slight fear is associated with notable responses.
